# Dengue-specific serotype related to clinical severity during the 2012/2013 epidemic in centre of Brazil

**DOI:** 10.1186/s40249-017-0328-9

**Published:** 2017-08-02

**Authors:** Benigno A. M. Rocha, Adriana O. Guilarde, Angela F. L. T. Argolo, Marianna Peres Tassara, Lucimeire A. da Silveira, Isabela C. Junqueira, Marília D. Turchi, Valéria C. R. Féres, Celina M. T. Martelli

**Affiliations:** 1 Institute of Tropical Pathology and Public Health, Federal University of Goiás, Goiânia, Brazil; 2 Faculty of Pharmacy, Federal University of Goiás, Goiânia, Brazil; 3 School of Nursing, State University of Goiás, Ceres, Brazil

**Keywords:** Dengue, Secondary infection, Severe dengue, Dengue type 4, Brazil

## Abstract

**Multilingual abstracts:**

Please see Additional file 1 for translations of the abstract into the five official working languages of the United Nations.

**Background:**

Currently, in Brazil, there is a co-circulation of the four dengue (DENV-1 to DENV-4) serotypes. This study aimed to assess whether different serotypes and antibody response patterns were associated with the severity of the disease during a dengue outbreak, which occurred in 2012/2013 in centre of Brazil.

**Methods:**

We conducted a prospective study with 452 patients with laboratory confirmed dengue in central Brazil, from January 2012 to July 2013. The clinical outcome was the severity of cases: dengue, dengue with warning signs, and severe dengue. The patients were evaluated at three different moments. Blood sampling for laboratory testing and confirmatory tests for dengue infection were performed. We performed a multinomial analysis considering the three categories of the dependent variable, as outlined above. The odds ratios (*OR*s) were calculated. A multinomial logistic regression model was applied for variables with a *P*-value <0.20. Statistical analysis was performed with STATA 12.0 software.

**Results:**

Four hundred fifty-two patients (452/632, 71.5%) were diagnosed with dengue. The dengue virus (DENV) serotypes were identified in 243 cases. DENV-4 was detected in 135 patients (55.6%), DENV-1 in 91 (37.4%), DENV-3 in 13 (5.3%), and DENV-2 in 4 (1.6%). Patients with the DENV-1 serotype were more prone to present with several clinical and laboratory features as compared with DENV-4 patients, including spontaneous bleeding (*P* = 0.03), intense abdominal pain (*P* = 0.004), neurological symptoms (*P* = 0.09), and thrombocytopenia (*P* = 0.01). Secondary infection was more predominant among DENV-4 cases (80.0%) compared with DENV-1 cases (62.3%) (*P* = 0.03). The univariate analysis showed that females (*OR* = 2.12; 95% *CI*: 1.44–3.13; *P* < 0.01) had a higher risk of having dengue with warning signs. The multinomial analysis showed that severe dengue cases with secondary infection had an adjusted *OR* of 2.80 (95% *CI*: 0.78–10.00; *P* = 0.113) as compared with dengue fever with primary infection when adjusted for age and sex.

**Conclusion:**

The current data show that 5.8% of patients recruited for treatment in healthcare centres and hospitals during the study period had severe dengue. DENV-4 was the predominant serotype, followed by DENV-1, in a large outbreak of dengue in central Brazil. Our findings contribute to the understanding of clinical differences and immune status related to the serotypes DENV-1 and DENV-4 in central of Brazil.

**Electronic supplementary material:**

The online version of this article (doi:10.1186/s40249-017-0328-9) contains supplementary material, which is available to authorized users.

## Background

The etiologic agents of dengue fever and dengue hemorrhagic fever (DF/DHF) are four serotypes: dengue virus (DENV)-1, DENV-2, DENV-3, and DENV-4, part of the dengue complex of the genus *Flavivirus* [1]. Dengue is a vector-borne viral disease considered to be a global public health issue due to an increasing incidence and its potential to cause epidemics and/or continuous viral circulation in most urban areas in tropical and subtropical regions of the world. In 2010, approximately 390 million dengue-infected individuals and 20,000 deaths were estimated worldwide [2–4].

In Brazil, dengue has been reported yearly since 1986 being widespread from the Atlantic coastal area to other Brazilian macroregions. In 2000, 60% of the dengue cases reported in South America occurred in Brazil [5]. The Brazilian Surveillance System registered at least four dengue epidemics in 2002, 2008, 2010, and 2013, with a predominance with a predominance of the serotypes DENV-3, DENV-2, DENV-1 and DENV-4 in each year, respectively. Currently there is a co-circulation of the four dengue serotypes after DENV-4 was reintroduced in 2010 [6–9].

Dengue presents with a range of symptoms, ranging from asymptomatic through to mild infection to severe illness with life-threatening outcomes*.* According to the disease progression, there are three clinical phases: the initial febrile phase from 1 to 3 days after the onset of symptoms, followed by the critical phase (4–7 days), and recovery or death. The majority of symptomatic cases progress to DF, considered to be the mild form of the disease.

Clinical classification of dengue has been a matter of extensive discussion in literature [10–15]. The current classification reflects the severity of the clinical features namely DF, dengue with warning signs (DwS), and severe dengue (SD). It has been adopted by the World Health Organization (WHO) and the Brazilian Ministry of Health to guide clinical management [2, 16, 17]. The potential for increasing vascular permeability is the hallmark of severe disease progression [2, 18–20]. Other specific organ involvement such as skin, eye, musculoskeletal system, gastrointestinal tract, liver, kidney and genitourinary tract, heart, and respiratory system are part of the dengue clinical presentation [21–23].

As the four serotypes are considered antigenically related but distinct, the previous immune status of the infected individuals plays an important role in disease progression [24]. In fact, several potential individual risk factors are implicated in dengue severity such as age, gender, immune status related to previous heterologous DENV infection, and co-morbidities, among others [24–28]. Most of the current literature is from Southeast Asia, where the DENV has been circulating for longer time (several decades). In this sense, there is a greater opportunity for research due to the distinct epidemiologic scenarios related to virus circulation and the immunity of the population in many endemic regions [29–32].

In a previous study, we explored the effects of viremic levels of type of infection, primary and secondary, in relation to the severity of the disease in the adult population during a DENV-3 epidemic in early 2000 in central of Brazil [21]. Here, we present a clinical cohort of dengue patients recruited during a DENV-4 outbreak in 2013, which had the largest reported number of incident cases (2233 suspected cases per 100,000 inhabitants) at state level (Goiás, central Brazil) [33, 34]. This was the first time that a simultaneous co-circulation of the four dengue serotypes was detected regionally. This scenario represents an opportunity to explore the immune status of the population, serotypes, and other potential risk factors related to severe disease progression.

The aim of the current study was to assess whether different serotypes and antibody response patterns were associated with the severity of the disease during a dengue outbreak in 2012/2013 in central of Brazil.

## Methods

### Study design and setting

We recruited 632 clinically suspected dengue cases, out which 452 (71.5%) were laboratory confirmed dengue cases. We conducted a prospective study of these laboratory confirmed dengue patients recruited at three healthcare units and four hospitals in the city of Goiânia (1.4 million inhabitants; Instituto Brasileiro de Geografia e Estatística, 2013), central Brazil, from January 2012 through to July 2013. We recruited patients who attended dengue reference centres established by the Secretariat of Health to deal with the referral of patients during the dengue outbreak in a timely manner. All recruitment sites had clinical expertise and operational capability to provide day healthcare monitoring and intravenous fluid replacement for suspected dengue cases (see Additional file 2).

The study site is a dengue endemic region where dengue incidence peaks during the rainy season (December to March). The dengue virus circulating in this region since 1994, when the official surveillance system detected DENV-1 serotype. During the next two decades, other dengue serotypes were introduced regionally according to the following temporal sequence: DENV-2 (1998), DENV-3 (2002), and DENV-4 (2011). Currently, the four dengue serotypes co-circulate in central Brazil [6, 34, 35]. The incidence of dengue disease was approximately 4500 cases per 100,000 inhabitants in the study setting in 2013. This figure is the highest rate of incidence since dengue was detected in the region, in 1994.

### Eligibility criteria and follow-up procedures

Inclusion criteria were laboratory confirmed dengue cases. The virologic and serologic tests for dengue diagnosis were: NS1 antigen positive test and/or detection of serotypes by multiplex polymerase chain reaction (PCR) and/or immunoglobulin M (IgM) serologic positive result by antibody capture enzyme-linked immunosorbent assay (MAC-ELISA).

Exclusion criteria were clinically suspected dengue cases with communication impairment, residents living outside city boundaries, and those with restrictions to comply with follow-up procedures. We also excluded outpatients who did not remain in health facilities for clinical management or monitoring.

Independently of this study protocol, local clinicians were responsible for all management decisions relating to routine health attendance, following the official guidelines [16].

### Clinical outcome

The clinical outcome was the severity of dengue cases defined as DF*,* DwS, or SD [2]. Two infectious disease doctors classified the dengue cases at the end of the follow-up period.

### Screening procedures at baseline

We screened patients with clinically suspected dengue independently of their age. We examined outpatients who were receiving intravenous fluid replacement and hospitalized patients. After informed consent was given, trained researchers obtained demographic information and clinical history, and performed medical examinations using standard case report forms. In addition, we collected an initial blood sample for diagnostic laboratory confirmation *(t1)*.

### Follow-up

Follow-up visits were scheduled: during the early convalescent phase ≥7 days after the onset of symptoms *(t2*); and late convalescent phase at 30–45 days after the onset of symptoms *(t3)*. In addition to the clinical examination, we collected blood samples for dengue monitoring, adhering to the same intervals.

For outpatients, the duration of follow-up was the time lag between the first and last blood collections (*t2* or *t3*) performed during the convalescent phase of the disease. The duration of follow-up for hospitalized patients was defined as the period from the first medical visit to either the discharge date or death.

### Data collection

We collected sociodemographic characteristics, such as age, sex, socioeconomic status, education, previous dengue episodes, and key warning signs of illness (e.g., hypotension, intense abdominal pain, and significant bleeding), from the patients.

### Dengue classification

We used the current dengue classification guidelines recommended by the Dengue Control Program (Brazilian Ministry of Health, 2009), with the recommended WHO classification [2], as:

- DF: The disease may manifest as a nonspecific febrile syndrome including the presence of acute febrile illness and two of the following symptoms: headache, retro-orbital pain, myalgia, arthralgia, rash, or hemorrhagic manifestations.
- DwS: The patient may present with persistent and severe abdominal pain, persistent vomiting, fluid accumulation, mucosal bleeding, altered mental status, hepatomegaly, and progressive increase in hematocrit.
- SD: Defined by one or more of the following: (i) shock from plasma leakage, fluid accumulation with respiratory distress, or both, (ii) severe bleeding as evaluated by a clinician, or (iii) severe organ involvement; liver: aspartate aminotransferase (AST) or alanine aminotransferase (ALT) > 1000; central nervous system: impaired consciousness; and injury heart and other organs.

### Definition of variables

The first seven days after the onset of symptoms denote the acute phase of illness. We defined illness “day 1” as the day of the onset of symptoms. Patients receiving day care were those patients who stayed in the hospital for intravenous fluid replacement for 24 h. Ambulatory patients were patients who attended ambulatory care units and required clinical monitoring and/or intravenous fluid replacement for <24 h.

### Laboratory procedures

Blood samples (10 ml) were collected at the initial clinical visit and at follow-up visits. Samples were prepared and sera were cryopreserved according to biosafety guidelines in freezers at −20 °C for serological tests and −80 °C for molecular tests, at the research centre (Laboratory of Molecular Biology and Immunology of Infectious Diseases) of the Institute of Tropical Pathology and Public Health, Federal University of Goiás, central Brazil.

During the baseline and follow-up visits, unspecific laboratory tests were also performed for all eligible patients, including hematocrit, platelet, AST, ALT, and albumin. The reference values for normality were a serum AST level of 50 U/L, a serum ALT level of 41 U/L, and an albumin level of 3.5–5.0 g/dl. Tests were performed at the Rômulo Rocha Laboratory, Faculty of Pharmacy, Federal University of Goiás, independently of the laboratory routine procedures followed at the healthcare units.

#### Serological tests

Acute and convalescent-paired sera were tested using the Dengue IgM Capture ELISA (Panbio®, Brisbane, Australia) and Dengue IgG Indirect ELISA (Panbio®, Brisbane, Australia) commercial kits. A NS1 antigen test was performed at baseline (Platelia™, Bio-Rad, California, USA). All tests were conducted according to the manufacturers’ instructions.

#### Molecular tests

The serotypes were identified by protocol, which includes viral RNA extraction using the QiAamp® Viral RNA Mini Kit (QIAGEN Inc., Germantown, MD, EUA). The complementary DNA (cDNA) was obtained by reverse transcription (RT) at 40 °C for 60 min with 10 μl of viral RNA and a mix containing 10 mL random primer and 50 U/ μl reverse transcriptase (Applied Biosystems™ High-Capacity cDNA Reverse Transcription Kit, Applied Biosystems, MA, USA).

The viral typing was performed by cDNA amplification using consensus primers (D1 and D2) for the four serotypes of DENV, the complementary sequences of the genes encoding the C and prM proteins. For the amplification of viral RNA the thermocycler SwiftTM Maxi Thermal Cycler (ESCO Technologies, OR, USA) was used. Then, the semi-nested specific primers TS1, TS2, TS3, and TS4 were used to detect each serotype of DENV, from 1 to 4 Lanciotti et al. [36]. The bands were visualized on 1.2% agarose gel using a transilluminator (UV Transilluminator, MUV Major Science, CA US).

A primary infection was defined by detecting IgM antibodies and/or nucleic acid and/or antigen NS1 in acute serum samples and seroconversion of IgG in the convalescent phase. A secondary infection was defined by detecting IgG antibodies in acute serum samples of patients with laboratory confirmed dengue.

### Data analysis

Initially, we applied descriptive statistics to assess the distribution of the variables in order to characterize the study population. The chi-square test and *t*-test were applied in order to verify statistical differences between categorical or continuous variables, respectively. We performed a multinomial analysis taking into account the three categories of the dependent variables (DF, DwS, SD) to calculate the association between the outcomes and the independent variables, considering DF as the reference. The odds ratios (*OR*s), with their respective 95% confidence intervals (95% *CI*s), and the *P*-values (chi-square test) were calculated. Variables that presented with a *P* < 0.20 in association with the outcome were eligible for the multivariate analysis. We applied a multinomial logistic regression model to adjust *OR*s for sex and age as a continuous variable. The software used for the multinomial analysis was STATA version 12.0 (StataCorp LLC, TE, USA).

### Ethical considerations

This study was approved by the Ethics Committee on Research of the Aggeu Magalhães Research Center (FIOCRUZ-PE) (No. 24/11) and the review board of each institution. Patients gave informed consent or when less than 18 years old this was given by the parents or guardians.

## Results

**Fig. 1:**
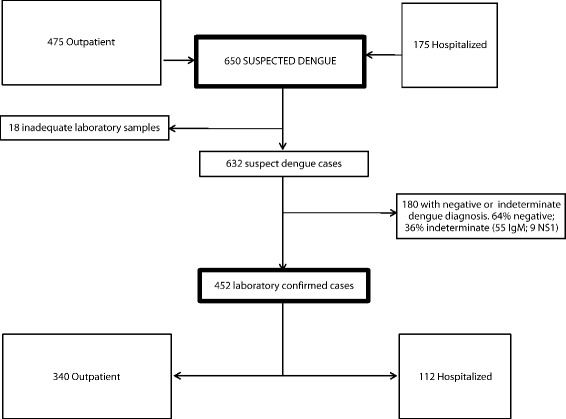
Flowchart showing suspected dengue cases recruited for the study

Of the 632 symptomatic dengue cases clinically and laboratorially screened at baseline, 452 (71.5%) were diagnosed as dengue confirmed by specific serology and/or viral detection (see Fig. 1). Table 1 presents the clinical and epidemiological data according to case ascertainment. In both groups (dengue confirmed and symptomatic cases), the majority of patients (~ 80%) were adult and approximately half were females. Approximately 75% of the patients were recruited in ambulatory settings and reported previous medical visits during the acute phase of the disease. Adult population comorbidity was similar between the groups.

**Table 1:** Clinical and epidemiological characteristics of 632 suspected dengue cases according to laboratory confirmation, recruited in Central Brazil, 2012 and 2013

Characteristics	Laboratory confirmed^a^ dengue cases (%)	Unconfirmed dengue cases (%)	*P* ^*b*^
Suspected cases dengue	452 (71.5)	180 (28.5)	–
Mean age (dp)	35.7 (17.5)	34.8 (16.6)	0.546
Age group (years)			
≤ 1	2 (0.4)	3 (1.7)	0.288
2–15	48 (10.6)	15 (8.3)	0.371
16–39	233 (51.5)	100 (55.6)	0.320
40–59	118 (26.1)	44 (24.4)	0.629
≥ 60	51 (11.3)	18 (10.0)	0.619
Gender			
Female	235 (52.0)	88 (48.9)	0.481
Male	217 (48.0)	92 (51.1)	0.481
Health care setting^c^			
Ambulatory	340 (75.2)	126 (70.0)	0.178
Hospital	112 (24.8)	54 (30.0)	0.178
Previous medical visit	142 (31.4)	64 (31.9)	0.316
Comorbidity^d^	115 (25.4)	48 (29.4)	0.751
Hypertension	82 (18.1)	30 (16.7)	0.661
Diabetes	25 (5.5)	11 (6.1)	0.776
Asthma	14 (3.1)	8 (4.4)	0.404
Chronic renal failure	8 (1.8)	2 (1.1)	0.549
Others^c^	15 (3.3)	11 (7.2)	0.669
Deaths	1 (0.2)	1 (0.6)	0.487

^a^laboratory confirmed dengue cases were positive by at least one of the test (RT-PCR and/or NS1 and/or IgM).

^b^ *χ* ^2^ test

^c^considering 540 adult patient (≥ 18 years).

^d^lupus erythematosus; cancer; AIDS; transplantation and hepatitis B or C

**Fig. 2:**
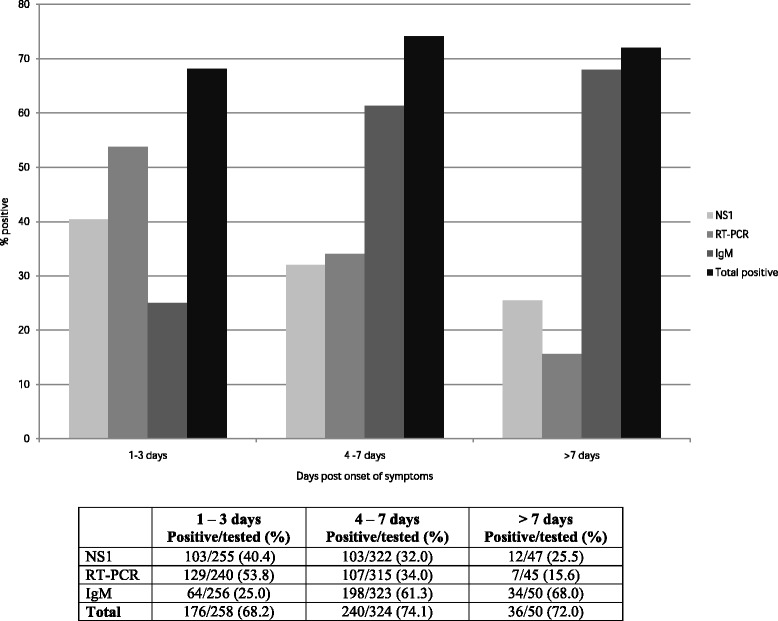
Laboratory diagnosis of symptomatic dengue cases according to days between the sample collection and the onset of symptoms during the 2012/2013 epidemic in the city of Goiânia, central Brazil

At baseline, the RT-PCR (53.8%) and NS1 (40.4%) tests yielded higher positive results compared with IgM serology (25%) during days 1–3 after the onset of symptoms. In contrast, higher frequencies of IgM positive results (68%) were detected seven days after the onset of symptoms versus RT-PCR (15.6%) and/or NS1 (25.5%) results (see Fig. 2).

Table 2 presents the main clinical and laboratory findings of confirmed dengue patients stratified by age group. At baseline, the most frequent symptoms reported were fever (100%), headache (~ 80%), and prostration (~ 90%), and these were similar among the age groups. Approximately 65% of patients presented with a cutaneous rash and approximately half reported vomiting at baseline. During the course of the illness, higher frequencies of signs and symptoms were observed among children, such as spontaneous bleeding (47.6% versus 26.6% among adults). In addition, intensive abdominal pain, effusion, and ascites were predominant among children as compared to adults, and a statistically significant difference was observed between the age groups. Children had a higher frequency of primary infection (39.4% versus 22% among the adult population), which was a statistically significant difference.

**Table 2:** Clinical and laboratory findings of confirmed dengue patients estratified by age-group

Features	< 15 years *N* = 42 (%)	≥ 15 years *N* = 410 (%)	*P* ^a^
Signs and symptoms reported or observed during recruitment			
Age, years - median (min-max)	10.1 (0–14)	38.4 (15–83)	–
Female sex	25 (59.5)	210 (51.2)	0.305
Days of illness - median (min-max)	4 (2–8)	5 (1–13)	0.535^g^
Headache	36 (85.7)	335 (81.7)	0.519
Prostration	41 (97.6)	373 (91.0)	0.139
Rash	29 (69.0)	269 (65.6)	0.654
Vomit	23 (54.8)	175 (42.7)	0.133
Signs and symptoms reported or observed during the course of the disease			
Spontaneous bleeding	20 (47.6)	109 (26.6)	0.004
Gastrointestinal bleeding	3 (7.1)	22 (5.4)	0.631
Neurological alterations	1 (2.4)	24 (5.9)	0.346
Breathing difficulties	0	51 (12.4)	0.015
Icterus	1 (2.4)	17 (4.1)	0.576
Intense abdominal pain	12 (28.6)	66 (16.1)	0.042
Hepatomegaly	3 (11.1)	24 (5.9)	0.737
Effusions and Ascites	4 (9.5)	8 (2.0)	0.004
Main laboratory results during the course of the disease			
Hemoconcentration^b^	12 (29.3)	77 (19.6)	0.146
Leukopenia (< 4000 cel/ml)^c^	20 (50.0)	230 (58.4)	0.223
Thrombocytopenia (< 100.000 cel/ml)^d^	9 (22.0)	123 (31.1)	0.307
AST (> 1000)^e^	0	0	–
ALT (> 1000)^f^	0	0	–
Clinical classification			
Dengue Fever	12 (28.6)	176 (42.9)	0.075
Dengue with Warning Signs	27 (64.3)	211 (51.5)	0.103
Severe Dengue	3 (7.1)	23 (5.6)	0.307
Antibody response pattern^h^			
Primary	13 (39.4)	67 (22.7)	0.034
Secondary	20 (60.6)	228 (77.3)	0.034

Hemoconcentration (children >44%, adult >48%)

^a^ *χ* ^2^ test

^b^432 results available

^c^435 results available

^d^433 results available

^e^416 results available

^f^415 results available

^g^Mann-Whitney Test

^h^328 analysed

Of the 452 patients surveyed, they were classified as Dengue Fever 188 (41.6%), Dengue with Alert Signs 238 (52.6%) and Severe Dengue 26 (5.8%). The DENV serotypes were identified in 243 (53.8%) out of 452 patients, tested by RT-PCR. DENV-4 was the predominant serotype, detected in 135 (55.6%) patients, followed by DENV-1 in 91 (37.4%) patients. DENV-3 and DENV-2 were detected in 13 (5.3%) and 4 (1.6%) patients, respectively (see Table 3).

**Table 3:** Clinical and epidemiological characteristics of 452 laboratory confirmed dengue cases, Central Brazil, 2012 and 2013

Parameters	Dengue Fever *N* = 188 (%)	Dengue with Warning Signs *N* = 238 (%)	Severe Dengue *N* = 26 (%)
Gender			
Female	78 (41.5)	143 (60.1)	14 (53.8)
Age group (years)			
< 1	1 (0.5)	1 (0.5)	0
2 a 14	11 (5.8)	26 (10.9)	3 (11.5)
15 a 39	108 (57.5)	123 (51.6)	10 (38.5)
40 a 59	44 (23.4)	63 (26.5)	11 (42.3)
≥ 60	24 (12.8)	25 (10.5)	2 (7.7)
Comorbidity	46 (24.5)	60 (25.2)	9 (34.6)
Antibody response pattern			
Primary	38 (27.3)	40 (23.0)	2 (13.3)
Secondary	101 (72.7)	134 (77.0)	13 (86.7)
Serotypes^a^			
DENV-1	35 (18.6)	47 (19.7)	9 (34.6)
DENV-2	2 (1.1)	2 (0.8)	0
DENV-3	3 (1.6)	10 (4.2)	0
DENV-4	63 (33.5)	72 (30.3)	0
Undetectable	75 (39.9)	98 (41.2)	15 (57.7)
Not done	10 (5.3)	9 (3.8)	2 (7.7)

^a^Serotypes determined by reverse-transcription polymerase chain reaction (RT-PCR)

Among the 26 cases of SD, nine had the DENV serotypes identified and all were infected with DENV-1; comprising five males and four females, aged seven to 57 years (median: 43 years). Descriptive analyses by serotype showed similar age and gender distributions of the patients infected with serotypes DENV-1 and DENV-4; the majority of patients were adults and approximately half were females. Each serotype included a large range of clinical forms and biological variations (data not shown).

Patients with a detectable DENV-1 serotype were more prone to present with several clinical and laboratory features as compared with DENV-4 patients, including spontaneous bleeding (DENV-1: 33.0% versus DENV-4: 20.0%; *P =* 0.03); intense abdominal pain (DENV-1: 29.7% versus DENV-4: 14.1%; *P =* 0.004); neurological symptoms (DENV-1: 6.7% versus DENV-4: 2.2%; *P =* 0.09); and thrombocytopenia (DENV-1: 33.7% versus DENV-4: 18.2%; *P =* 0.01). The immune status measured by primary or secondary infections were available for 202 patients (DENV-1 or DENV-4). Secondary infection was more predominant among DENV-4 cases (80.0%) compared with DENV-1 cases (62.3%), a statistically significant difference between the serotypes (*P =* 0.03).

Table 4 presents the results of the multinomial analysis (the association between the antibody response pattern, serotype, and severity of disease taking DF as a reference). The univariate analysis showed that females were at a higher risk of having DwS (*OR* = 2.12; 95% *CI*: 1.44–3.13; *P* < 0.01) in comparison with DF patients. Females classified as having SD did not differ from the reference. Adult patients (≥ 15 years old) had an *OR* of 0.53 (95% *CI*: 0.26–1.08; *P =* 0.082) compared to children with DF. Comorbidity was not associated with the severity of the disease. Patients classified as having DwS and that mounted secondary infection were found to be not at risk (*OR* = 1.03; 95% *CI*: 0.66–1.60; *P =* 0.890) compared with DF patients with primary infection. In addition, SD cases with secondary infection had an *OR* of 2.63 (95% *CI*: 0.74–9.30; *P =* 0.134). Results of DENV-1 and DENV-4 were presented for DwS versus FD, as few cases of DENV-2 and DENV-3 were detected in this study. The multinomial analysis showed that SD cases with secondary infection had an adjusted *OR* of 2.80 (95% *CI*: 0.78–10.00; *P =* 0.113) compared with DF patients with primary infection, when adjusted for age and sex (see Table 5).

**Table 4:** Multinomial analysis of the association between antibody response pattern, serotype and severity of dengue disease as outcome

Parameters	Dengue Fever	Dengue with Warning Signs			Severe Dengue		
	*N* = 188 (%)	*N* = 238 (%)	*OR* (95% *CI*)	*P*-value	*N* = 26 (%)	*OR* (95% *CI*)	*P*-value
Gender							
Female	78 (41.5)	143 (60.1)	2.12 (1.44–3.13)	0.000	14 (53.8)	1.64 (0.72–3.75)	0.236
Age group (years)							
< 15	12 (6.4)	27 (11.3)	Reference	–	3 (11.5)	Reference	–
≥ 15	176 (93.6)	211 (88.7)	0.53 (0.26–1.08)	0.082	23 (88.5)	0.52 (0.14–1.99)	0.342
Comorbidity	46 (24.5)	60 (25.2)	1.04 (0.67–1.62)	0.860	9 (34.6)	1.63 (0.68–3.92)	0.271
Antibody response pattern^a^							
Primary	53 (28.2)	62 (26.1)	Reference	–	3 (11.5)	Reference	–
Secondary	121 (64.4)	146 (61.3)	1.03 (0.66–1.60)	0.890	18 (69.2)	2.63 (0.74–9.30)	0.134
Serotype^b^							
DENV-1	35 (19.7)	47 (19.7)	Reference	–	9 (34.6)	NA	–
DENV-4	63 (35.4)	72 (30.3)	0.85 (0.49–1.48)	0.568	0	NA	–

Reference for the multinomial analysis: dengue fever

*NA* Not applicable

^a^49 patients not evaluated

^b^Serotypes determined by reverse-transcription polymerase chain reaction (RT-PCR) and 226 patients not evaluated

**Table 5:** Multinomial analysis adjusted by age and sex of the association between antibody response pattern, serotype and severity of dengue disease as outcome

Parameters	Dengue with Warning Signs		Severe Dengue	
	*OR*_Adj_ (95% *CI*)	*P*-value	*OR*_Adj_ (95% *CI*)	*P*-value
Comorbidity	1.20 (0.72–2.00)	0.479	1.74 (0.64–4.73)	0.279
Antibody response pattern				
Primary	Reference	–	Reference	–
Secondary	1.20 (0.76–1.89)	0.433	2.80 (0.78–10.0)	0.113
Missing	1.93 (0.92–4.09)	0.083	6.23 (1.32–29.4)	0.021
Serotype^a^				
DENV-1	Reference	–	NA	–
DENV-4	0.80 (0.45–1.41)	0.444	NA	–
Not evaluated	0.95 (0.56–1.62)	0.856	NA	–

^a^Serotypes determined by reverse-transcription polymerase chain reaction (RT-PCR)

## Discussion

Our results show differences in the clinical features of dengue patients infected during a large DENV-4 outbreak in central of Brazil. Children presented higher frequencies of several warning signs of disease severity such as spontaneous bleeding, intensive abdominal pain, and neurological symptoms when compared to adults. Secondary infections were more prone to occur in the adult population, however, more than 60% of the children and almost 80% of the adult patients in this study were found to have a previous dengue infection, highlighting the high DENV circulation in the region.

Few studies have compared clinical features and laboratory abnormalities in the pediatric age group and adults in Brazil [37, 38]. A study conducted in the same region in 2005 showed that secondary infection was not a predictor of severe clinical manifestation in adults infected with the DENV-3 serotype [21].

In a prospective clinical study conducted in the same city in 2000, we found that mild cases of dengue were predominant among adults [21]. In this study, we classified the majority of the pediatric and adult patients as having DwS, followed by DF, and a few cases of SD. This distribution reflects the clinical characteristics of dengue patients treated at reference day clinics and hospitals during the 2012/2013 epidemic. It does not resemble the entire cohort of dengue patients, as most of the cases were milder cases classified as DF, as according to the surveillance system (SINAN, 2012/2013).

It is important to note that all patients classified as having SD were infected with DENV-1 and none were infected with DENV-4. However, we are aware that our sample size relating to the severe form of the disease is too small to draw a conclusion. Interestingly, the historical data outlined by Hastead in the early decades of dengue epidemics regarding differences in clinical manifestations of DENV-1 and DENV-4 serotypes in Southeast Asia similarly describe DENV-1 as being more prone to cause severe cases as compared with DENV-4 [39].

In our study, we found a high percentage of undetectable viremia among severe cases. Dengue patients may progress to severe disease during the defervescence period, which is the period of hospital admission and recruitment that is beyond the viremic period [21]. We are aware that this could represent a potential selection bias in the recruitment of severe cases.

One of the strengths of this study was the recruitment of dengue patients approximately two years after the introduction of the DENV-4 serotype in central Brazil. In this context, it is likely that the majority of the population in the study area was naïve to DENV-4, which explains the current outbreak with the predominance of the DENV-4 circulation. In fact, DENV-4 had been previously isolated in nine patients in the neighbouring state of Mato Grosso do Sul in 2012. The authors had warned about the potential for outbreaks due to the introduction of the DENV-4 serotype in a susceptible population to this serotype in central Brazil [40].

Another strength of our study is that we recruited patients in several ambulatories and hospital settings within the region. However, our study population included only patients living in one city and the results may not be generalizable to rural areas or other regions of the country.

Comparison of clinical manifestations, antibody response patterns, and severity of the disease were restricted to the DENV-4 and DENV-1 serotypes, as few patients had detectable DENV-2 or DENV-3 serotypes in this study. These findings are concordant with the official laboratory system in charge of DENV surveillance regionally. It is interesting that the DENV-2 serotype had not been predominantly registered by the viral surveillance system in the last two decades in the study area [35]. We are aware that according to the period when the blood samples for serological tests (IgM or IgG) were collected, this may yield negative or positive results, which could lead to the misclassification of primary and secondary infections.

## Conclusions

In summary, the present study shows the incidence of SD among pediatric and adult patients in the first registered DENV-4 outbreak in central Brazil. To our knowledge, this is the first prospective clinical study to compare DENV-1 and DENV-4 patients in relation to antibody response patterns and severity of the disease. Our findings contribute to the understanding of clinical differences and immune status related to the serotypes DENV-1 and DENV-4 in central Brazil.

## Additional file

Additional file 1: Multilingual abstracts in the five official working languages of the United Nations. (PDF 644 kb)
Additional file 2: Association between clinical or laboratory markers and dengue serotypes, Central Brazil, 2012 and 2013. (DOCX 13 kb)

## Abbreviations

ALT: Alanine aminotransferase
AST: Aspartate aminotransferase
cDNA: 23 complementary DNA
cDNA: Deoxyribonucleic acid
*CI*: Confidence interval
CNS: Central nervous system
DENV: Dengue virus
DENV: Virus dengue
DF: Dengue fever
DF: Fever dengue
DHF: Hemorrhagic dengue fever
DwC: Dengue with warning signs
ELISA: Enzyme-linked immunosorbent assay
IBGE: Intituto Brasileiro de Geografia e Estatística
Ig: Immunoglobulin
IgG: Immunoglobulin G
IgM: Immunoglobulin M
NS1: Non-structural protein-1
OR: Odds ratio
PCR: Polymerase chain reaction
RNA: Ribonucleic acid
RT: Reverse transcription
RT-PCR: Reverse transcription-polymerase chain reaction
SD: Severe dengue
STATA: Data analysis and statistical software
WHO: World Health Organization
