# Supplementary material for: Dengue-specific serotype related to clinical severity during the 2012/2013 epidemic in centre of Brazil
Source: Infect Dis Poverty. 2017 Aug 2;6:116. doi: 10.1186/s40249-017-0328-9 (PMC5540539; doi:10.1186/s40249-017-0328-9)
Supplement: Additional file 1: — Multilingual abstracts in the five official working languages of the United Nations. (PDF 644 kb) [file 40249_2017_328_MOESM1_ESM.pdf]

Translation of the abstract into the five official working languages of the United Nations

نَمَطٌ مَصْلِيّ خاص بحمى الضنك يتعلق بالشدة السريرية خلال وباء 2013/2012 في وسط البرازيل

بنينو أ. م. روشا، أدريانا و جيلاردي، أنجيلا ف. ل. ت أرجولو، ماريانا بيريز تاسارا، لوسيميري أ دا سيلفيرا، ايزابيلا س خوانكويرا، ماريليا د تورتشي، فاليريا جيم ر فيريس، سيلينا م ت مارتيلي

ملخص
خلفية: في الوقت الراهن، في البرازيل، تنتشر الأنماط المصلية لحمى الضنك الأربعة (DENV-1 إلى DENV-4) معا. هدفت هذه الدراسة إلى تقييم ما إذا كانت أنماط استجابة الأنماط المصلية والأجسام المضادة المختلفة مرتبطة مع شدة المرض خلال تفشي حمى الضنك، الذي وقع في 2013/2012 في وسط البرازيل.
الأسلوب: أجرينا دراسة استطلاعية مع 452 مريضا مع تأكد إصابتهم معمليا بحمى الضنك في وسط البرازيل، من يناير 2012 إلى يوليو 2013. وكانت النتيجة السريرية لشدة الحالات: حمى الضنك، حمى الضنك مع علامات إنذار، حمى ضنك شديدة. وجرى تقييم المرضى في ثلاث أوقات مختلفة. أخذت عينات من الدم لفحصها في المختبر وإجراء اختبارات لعدوى حمى الضنك. أجرينا تحليلا متعدد الحدود واضعين في الاعتبار الفئات الثلاثة للمتغير التابع، على النحو المبين أعلاه. تم حساب نسب الأرجحية. تم تطبيق نموذج الانحدار اللوجستي متعدد الحدود للمتغيرات مع قيمة-P <0.20. تم إجراء التحليل الإحصائي باستخدام برنامج STATA 12.0.
النتائج: تم تشخيص أربعمائة واثنان وخمسون مريضا (632/452، 71.5٪) مصابين بحمى الضنك. وقد تم التعرف على الأنماط المصلية لفيروس حمى الضنك (DENV) في 243 حالة. تم الكشف عن DENV-4 في 135 مريضا (55.6٪)، DENV-1 في 91 (37.4٪)، DENV-3 في 13 (5.3٪)، وDENV-2 في 4 (1.6٪). وكان المرضى الذين يعانون من النمط المصلي DENV-1 أكثر عرضة لإظهار العديد من الخواص السريرية والمعملية بالمقارنة مع مرضى DENV-4، بما في ذلك النزف التلقائي (P = 0.03)، ألم شديد في البطن (ع = 0.004)، وأعراض عصبية (P = 0.09)، ونقص الصفيحات (P = 0.01). كانت العدوى الثانوية سائدة أكثر بين حالات DENV-4 (80.0٪) مقارنة مع حالات DENV-1 (62.3٪) (P= 0.03). أظهر التحليل أحادي المتغير أن الإناث (OR = 2.12، 95٪ CI: 1.44-3.13؛ P<0.01) عرضة أكثر للإصابة بحمى الضنك مع علامات إنذار. وأظهر التحليل متعدد الحدود أن لحالات حمى الضنك الشديدة المقترنة بعدوى ثانوية OR معدل 2.80 (95٪ CI: 0.78-10،؛ P= 0.113) بالمقارنة مع حمى الضنك المقترنة بعدوى أولية عند تعديله بالنسبة للعمر والجنس.
الخلاصة: تظهر البيانات الحالية أن 5.8٪ من المرضى الذين خضعوا للعلاج في مراكز الرعاية الصحية والمستشفيات خلال فترة الدراسة عانوا من حمى ضنك شديدة. كان النمط المصلي DENV-4 هو السائد، يليه DENV-1، عند حدوث حمى الضنك في وسط البرازيل. وتساهم النتائج التي توصلنا إليها في فهم الاختلافات السريرية والحالة المناعية المرتبطة بالمصليين DENV-1 وDENV-4 في وسط البرازيل.

Translated from English version into Arabic by Hassan Adam, through

TRANSLATORS
WITHOUT BORDERS

2012/2013 巴西中部地区登革热流行中特异性血清型与临床严重程度的关系

Benigno A. M. Rocha, Adriana O Guilarde, Angela F. L. T. Argolo, Marianna Peres Tassara, Lucimeire A. da Silveira, Isabela C Junqueira, Marília D Turchi, Valéria C. R. Féres and Celina M T Martelli

摘要
引言：目前，在巴西有四种(DENV- 1- DENV-4) 登革血清型。本研究的目的是评估 2012 及 2013 年在巴西中部爆发的登革热中不同的血清型和抗体反应模式与疾病的严重程度的是否存在关联。
方法：我们从 2012 年 1 月至 2013 年 7 月在巴西中部开展了针对 452 名确诊病例的前瞻性调查研究。临床结果按病例严重程度分为：登革热、有警示症状的登革热和重症登革热。在 3 个节点对病人进行评估。对血样分别进行实验室检测和确诊检测。根据上述的 3 个因变量，我们对结果采取多项分析。我们计算了优势比(*OR*s)，并对变量以 *P*-value < 0.20 采用多项逻辑回归模型进行计算，统计软件采用 STATA 12.0 软件包。
结果：本研究共确诊 452 名登革热病例（452/632, 71.5%），并且在 243 名病例中确认了血清型。135 名病例为 DENV-4 血清型（55.6%），91 名病例为 DENV-1 血清型（37.4%），13 名

病例为 DENV-3 血清型（5.3%）， 4 名病例为 DENV-2 血清型（1.6%）。与 DENV-4 血清型的病例相比，DENV-1 血清型的病例更容易具有严重的临床和实验室症状，包括自发性出血（*P* = 0.03），严重腹痛（*p*=0.004），神经症状（*P* = 0.09）和血小板减少症（*P* = 0.01）。与 DENV-1 血清型病例（62.3%）相比，DENV-4 血清型的病例(80.0%)更容易出现二次感染（*P* = 0.03）。单变量分析显示女性患登革热后更容易有警示症状（*OR* = 2.12; 95% *CI*: 1.44 – 3.13; *P* < 0.01）。多项逻辑回归显示与初患病例相比，二次感染的重症登革热患者的校正优势比为 2.80（以性别和年龄进行校正）（95% *CI*: 0.78 – 10.0; *P* = 0.113）。
结论：目前的数据显示卫生中心和医院在研究期间招募的病人中有 5.8%为重症登革热患者。在巴西这次的主要的流行中，DENV-4 血清型为主要的血清型，其次为 DENV-1。我们的研究发现将有助于理解巴西中部地区 DENV-1 和 DENV-4 血清型的临床差异和免疫状态。

Translated from English version into Chinese by Men-Bao Qian, through

CHINESE CENTER FOR DISEASE CONTROL AND PREVENTION
NATIONAL INSTITUTE OF PARASITIC DISEASES

Sérotype spécifique à la dengue lié à la sévérité clinique au cours de l'épidémie de 2012/2013 au centre du Brésil

Benigno A. M. Rocha, Adriana O Guilarde, Angela F. L. T. Argolo, Marianna Peres Tassara, Lucimeire A. da Silveira, Isabela C Junqueira, Marília D Turchi, Valéria C. R. Féres, Celina M T Martelli

Résumé

Contexte : nous constatons actuellement au Brésil une co-circulation de quatre sérotypes de la dengue (DENV-1 à DENV-4). La présente étude sert à déterminer si les différents sérotypes et modèles de réponse immunitaire étaient associés à la sévérité de la maladie au cours d'une épidémie de dengue qui s'est produite de 2012 à 2013 au centre du Brésil.
Méthodes : nous avons mené une étude prospective auprès de 452 patients atteints d'une dengue confirmée par des analyses de laboratoire au centre du Brésil de janvier 2012 à juillet 2013. Le résultat clinique correspondait à la sévérité des cas : dengue, dengue associée à des signes d'avertissement et dengue sévère. Les patients ont été évalués à trois moments différents. Des prélèvements sanguins à des fins d'analyses de laboratoire et de tests de confirmation d'une infection par la dengue ont été réalisés. Nous avons réalisé une analyse multinomiale en tenant compte des trois catégories de la variable dépendante, comme souligné ci-dessus. Les rapports de cotes (*OR*) ont été calculés. Un modèle de régression logistique multinomiale a été appliqué pour des variables affichant une valeur *P* < 0,20. Une analyse statistique a été réalisée avec le logiciel STATA 12.0.
Résultats : la dengue a été diagnostiquée chez 452 patients (452/632, 71,5 %). Des sérotypes du virus de la dengue (DENV) ont été identifiés dans 243 cas. Le DENV-4 a été détecté chez135 patients (55,6 %), le DENV-1 chez 91 patients (37,4 %), le DENV-3 chez 13 patients (5,3 %) et le DENV-2 chez 4 patients (1,6 %). Les patients porteurs du sérotype DENV-1 étaient plus susceptibles de présenter plusieurs caractéristiques cliniques et de laboratoire par rapport aux patients porteurs du DENV-4, notamment des saignements spontanés (*P* = 0,03), d'intenses douleurs abdominales (*p*=0,004), des symptômes neurologiques (*P* = 0,09) et une thrombocytopénie (*P* = 0,01). L'infection secondaire était plus prédominante parmi les cas de DENV-4 (80,0 %) par rapport aux cas de DENV-1 (62,3 %) (*P* = 0,03). L'analyse univariée a démontré que les femmes (*OR* = 2,12 ; *IC* à 95 % : 1,44 – 3,13 ; *P* < 0,01) affichaient un risque plus élevé de contracter la dengue associée à des signes d'avertissement. L'analyse multinomiale a démontré que des cas de dengue sévère présentant une infection secondaire affichaient un *OR* de 2,80 (*IC* à 95 % : 0,78 – 10,0 ; *P* = 0,113) par rapport aux cas de dengue présentant une infection primaire lors d'un ajustement en fonction de l'âge et du sexe.
Conclusion : les données actuelles démontrent que 5,8 % des patients recrutés en vue d'être traités au sein de centres de soins de santé et d'hôpitaux au cours de la période de l'étude étaient atteints d'une dengue sévère. Le DENV-4 était le sérotype prédominant, suivi du DENV-1, dans le cadre d'une large épidémie de dengue au centre du Brésil. Nos résultats contribuent à la compréhension

des différences cliniques et du statut immunologique liés aux sérotypes DENV-1 et DENV-4 au centre du Brésil.

Translated from English version into French by eric ragu, through

TRANSLATORS
WITHOUT BORDERS

Денге-специфический серотип, связанный с клинической тяжестью в течение 2012/2013 эпидемии в центре Бразилии

Benigno A. M. Rocha, Adriana O Guilarde, Angela F. L. T. Argolo, Marianna Peres Tassara, Lucimeire A. da Silveira, Isabela C Junqueira, Marília D Turchi, Valéria C. R. Féres, Celina M T Martelli

Реферат
Фон: В настоящее время, в Бразилии есть совместное обращение четырех денге (DENV-1 в DENV-4) серотипов. Цель данного исследования состоится в том, что связанны ли разные серотипы и узоры иммунного ответа с тяжестью заболевания во время вспышки лихорадки денге, которая произошла в 2012/2013 в центре Бразилии.
Методы: Мы провели проспективное исследование с 452 пациентами с лабораторно подтвержденным денге в центральной части Бразилии с января 2012 по июль 2013 года. Клинический результат был тяжестью случаев: денге, денге с предупреждающими знаками, и тяжелым денге. Пациенты были оценены в трех различных моментах. Проводили забор крови для лабораторного тестирования и подтверждающие тесты на инфекции лихорадкаи. Мы провели полиномиальный анализ, чтобы рассматривать три категории зависимой переменной, как описано выше. Коэффициенты шансов (*OR*s) рассчитывалось. Мультиномиальная логистическая регрессия применяется для переменных *P*-value < 0.20. Статистический анализ проводился с STATA 12.0 программным обеспечением.
Результаты: Четыреста пятьдесят два пациента (452/632, 71.5%) была диагностирована лихорадкой денге. Серотипы вируса денге (DENV) были выявлены в 243 случаях. DENV-4 было выявлено у 135 больных (55.6%), DENV-1 у 91 (37.4%), DENV-3 у 13 (5.3%) и DENV-2 у 4 (1.6%). Пациенты с серотипом DENV-1 были более склонны к присутствующим с несколькими клинико-лабораторными особенностями по сравнению с больными с серотипа-4, включая спонтанное кровотечение (P = 0,03), интенсивные боли в животе (p=0,004), неврологическую симптоматику (P = 0,09) и тромбоцитопении (p = 0,01). Вторичная инфекция была еще преобладающей среди случаев DENV-4 (80.0%) по сравнению со случаями DENV-1 (62.3%) (P = 0,03). Однофакторный анализ показал, что женщины (*OR*= 2.12; 95% *CI:* 1.44 – 3.13; *P* < 0,01) имели высокий риск наличия денге с предупреждающими знаками. Полиномиальный анализ показал, что в тяжелых случаях лихорадки денге с вторичной инфекцией были скорректированы с *OR* = 2.80 (95% *CI*: 0,78 – 10.0; *P* = 0.113) по сравнению с лихорадкой денге с первичной инфекцией с учетом возраста и пола.
Заключени: Текущие данные показывают, что 5.8% пациентов, отобранных для лечения в медицинских центрах и больницах в течение периода исследования получили тяжелое денге. DENV-4 был доминирующим серотипом, с последующим DENV-1 в большой вспышке лихорадки денге в центральной Бразилии. Наши результаты вносят вклад в понимание различий клинического и иммунного статуса связаны с серотипами DENV-1 и DENV-4 в центральной Бразилии.

Translated from English version into Russian by Hao-Qi Zhang

Asociación entre serotipos específicos de dengue con la gravedad clínica de la enfermedad durante la epidemia de los años 2012/2013 en el centro de Brasil

Benigno A. M. Rocha, Adriana O Guilarde, Angela F. L. T. Argolo, Marianna Peres Tassara, Lucimeire A. da Silveira, Isabela C Junqueira, Marília D Turchi, Valéria C. R. Féres, Celina M T Martelli

Resumen

Antecedentes: En la actualidad, en Brasil co-circulan cuatro serotipos de dengue (DENV-1 a DENV-4). El presente estudio tuvo por objetivo evaluar si los distintos serotipos y los patrones de respuestas de anticuerpos se relacionaban con la gravedad de la enfermedad durante un brote de dengue que se produjo en el centro de Brasil en los años 2012/2013.

Métodos: Llevamos a cabo un estudio prospectivo con 452 pacientes con dengue confirmado por laboratorio en el centro de Brasil, entre enero de 2012 y julio de 2013. El resultado clínico fue la severidad de los casos: dengue, dengue con signos de alarma y dengue grave. Se evaluó a los pacientes en tres oportunidades distintas. Se tomaron muestras de sangre para las pruebas de laboratorio y las pruebas confirmatorias de dengue. Realizamos análisis multinomial teniendo en cuenta las tres categorías de la variable dependiente que se indicara anteriormente. Se calculó la razón de momios (RM). Se aplicó un modelo de regresión logística multinomial para las variables con un valor $P < 0{,}20$. Se realizó el análisis estadístico con el programa STATA 12.0.

Resultados: Cuatrocientos cincuenta y dos pacientes (452/632, 71,5%) fueron diagnosticados con dengue. En 243 casos se identificaron serotipos del virus del dengue (DENV). El DENV-4 se detectó en 135 pacientes (55,6%), el DENV-1 en 91 (37,4%), el DENV-3 en 13 (5,3%) y el DENV-2 en 4 (1,6%). Los pacientes con serotipo DENV-1 fueron más propensos a presentar cuadros clínicos y de laboratorio graves en comparación con los pacientes con DENV-4, incluido sangrado espontáneo ($P = 0{,}03$), dolor abdominal intenso (p=0,004), síntomas neurológicos ($P = 0{,}09$) y trombocitopenia ($P = 0{,}01$). Las infecciones secundarias fueron más predominantes en los casos con DENV-4 (80,0%) en comparación con los casos con DENV-1 (62,3%) ($P = 0{,}03$). El análisis univariado reveló que las mujeres (*RM*= 2,12; 95% *IC*: 1,44 – 3,13; $P < 0{,}01$) corrían mayor riesgo de presentar dengue con signos de alarma. El análisis multinomial reveló que los casos de dengue grave con infección secundaria tenían una *RM ajustada* de 2,80 (95% *IC*: 0,78 – 10,0; $P = 0{,}113$) en comparación con la fiebre de dengue con infección primaria cuando se ajustaba por edad y sexo.

Conclusión: La información actual indica que 5,8% de los pacientes reclutados para tratamiento en los centros de salud y hospitales durante el período de estudio padecían de dengue grave. El DENV-4 fue el serotipo predominante, seguido por el DENV-1, en un importante brote de dengue en el centro de Brasil. Nuestros hallazgos contribuyen a comprender las diferencias clínicas y los estados inmunes que se asocian con los serotipos DENV-1 y DENV-4 en el centro de Brasil.

Translated from English version into Spanish by Maria Alejandra Aguada, through

TRANSLATORS
WITHOUT BORDERS
