# Supplementary material for: Dengue-specific serotype related to clinical severity during the 2012/2013 epidemic in centre of Brazil
Source: Infect Dis Poverty. 2017 Aug 2;6:116. doi: 10.1186/s40249-017-0328-9 (PMC5540539; doi:10.1186/s40249-017-0328-9)
Supplement: Additional file 2: — Association between clinical or laboratory markers and dengue serotypes, Central Brazil, 2012 and 2013. (DOCX 13 kb) [file 40249_2017_328_MOESM2_ESM.docx]

| Parameters | Serotype | | *p^a^* |
| --- | --- | --- | --- |
|  | DENV-1 | DENV-4 |  |
|  | N= 91(%) | N=135 (%) |  |
| FD | 35 (38.5) | 63 (46.7) | 0.22 |
| DwS | 47 (51.6) | 72 (53.3) | 0.80 |
| SD | 9 (9.9) | 0 | *0.007* |
| Mean age (dp) | 36.4 (17.2) | 35.7 (15.9) | 0.78^b^ |
| Hospitalized | 11 (12.1) | 19 (14.1) | 0.666 |
| Gender |  |  |  |
| Female | 45 (49.5) | 69 (51.1) | 0.807 |
| Type infection^c^ |  |  |  |
| Primary | 29 (37.7) | 25 (20.0) | 0.003 |
| Secondary | 48 (62.3) | 100 (80.0) | 0.003 |
| Neurological symptoms | 6 (6.7) | 3 (2.2) | 0.09^d^ |
| Intense abdominal pains | 27 (29.7) | 19 (14.1) | *0.004* |
| Hepatomegaly | 5 (5.5) | 4 (3.0) | 0.34 |
| Thrombocytopenia | 29 (33.7) | 24 (18.2) | *0.01* |
| Spontaneous bleeding | 30 (33.0) | 27 (20.0) | *0.03* |
| DENV-1 = Virus dengue 1 e DENV-4 = Virus dengue 4  ^a^ *x^2^* Test  ^b^ *t*-test  ^c^ 202 case analized  ^d^  [Fisher's exact test](https://www.google.com.br/url?sa=t&rct=j&q=&esrc=s&source=web&cd=1&cad=rja&uact=8&ved=0CB4QFjAA&url=http%3A%2F%2Fen.wikipedia.org%2Fwiki%2FFisher%2527s_exact_test&ei=WzP2VPmQJonnsATJ6YG4Ag&usg=AFQjCNHVmzAGgVfgHyxEj3pY2w2-ohzCMw&sig2=VPAwD-iR59ROsOj29ovzkw)  FD = Fever Dengue  DwS = *Dengue with Warning Signs*  SD = Severe Dengue | | | |

Additional file1 Association between clinical or laboratory markers and dengue serotypes, Central Brazil, 2012 and 2013.
